# Unsachgemäße Systemtherapie bei schwerer atopischer Dermatitis – fatale Langzeitschäden

**DOI:** 10.1007/s00105-021-04922-1

**Published:** 2021-12-14

**Authors:** F. Abeck, N. Booken, S. Schneider

**Affiliations:** Present Address: Klinik und Poliklinik für Dermatologie und Venerologie, Universitätsklinikum Hamburg-Eppendorf, Martinistraße 52, 20246 Hamburg, Deutschland

**Keywords:** Atopische Dermatitis, Systemtherapie, Orale Glukokortikosteroide, Nebenwirkungen, Atopic dermatitis, Systemic therapy, Oral glucocorticoids, Adverse events

## Abstract

Die Therapiemöglichkeiten der schweren atopischen Dermatitis waren bis vor Kurzem sehr begrenzt und haben sich mit der Zulassung des ersten Biologikums Dupilumab Ende 2017 bis heute deutlich verbessert. Aktuell wurden mit dem Biologikum Tralokinumab sowie den Januskinase-Inhibitoren Baricitinib und Upadacitinib weitere neue Systemtherapeutika zugelassen. Dennoch gibt es Fälle, in denen es zur Nichtberücksichtigung moderner Behandlungsmöglichkeiten kommt, wie die Falldarstellung eines 28-jährigen Patienten mit schwerwiegenden Nebenwirkungen einer Langzeitbehandlung mit systemischen Glukokortikosteroiden zeigt. Neben der umfangreichen Abklärung der Folgeschäden, erfolgten die Einleitung einer leitliniengerechten Therapie mit Dupilumab sowie eine interdisziplinäre Zusammenarbeit mit Endokrinologen, Ophthalmologen, Osteologen und Ernährungsmedizinern.

## Abstract (en)

The therapeutic options for treatment of severe cases of atopic dermatitis have been limited until recently, but fundamentally improved with the approval of the first biological dupilumab at the end of 2017. With the biological tralokinumab and the Janus kinase inhibitors baricitinib and upadacitinib, further new systemic drugs have recently been approved. Nevertheless, there are cases in which modern treatment options are not taken into account, as shown by a 28-year-old patient with serious side effects from long-term treatment with systemic glucocorticoids. In addition to the extensive clarification of the consequential damage, guideline-based therapy with dupilumab was initiated as well as interdisciplinary cooperation with endocrinologists, ophthalmologists, osteologists and nutritionists.

## Anamnese

Wir berichten über einen 28-jährigen Patienten, bei dem seit dem ersten Lebensjahr eine atopische Dermatitis (AD) besteht. Die Familienanamnese ist positiv, da die Mutter ebenfalls von einer AD betroffen ist. Bei dem Patienten liegt zusätzlich ein allergisches Asthma bronchiale vor. Das Ekzem wurde seit 6 Jahren mit systemischen Glukokortikosteroiden behandelt. Die Dosierung wurde eigenständig durch den Patienten je nach Hautbefund und Juckreiz gewählt und habe stets zwischen 20 und 40 mg Prednisolon pro Tag betragen. Die Verschreibung der Glukokortikosteroide erfolgte jeweils durch unterschiedliche Hausärzte, die der Patient im Laufe der Zeit kontinuierlich wechselte. Der Patient berichtet von einer Gewichtszunahme von 36 kg in den letzten Jahren. Vor 4 Jahren sei eine Operation am rechten Auge aufgrund einer Katarakt erfolgt, worauf es im Anschluss zu einer Netzhautablösung mit folgender Erblindung rechts gekommen sei. Nach einem Sturz auf die rechte Hand vor 3 Wochen könne er zudem eine Stufenbildung am Handrücken tasten.

## Befund

**Figure 1:**
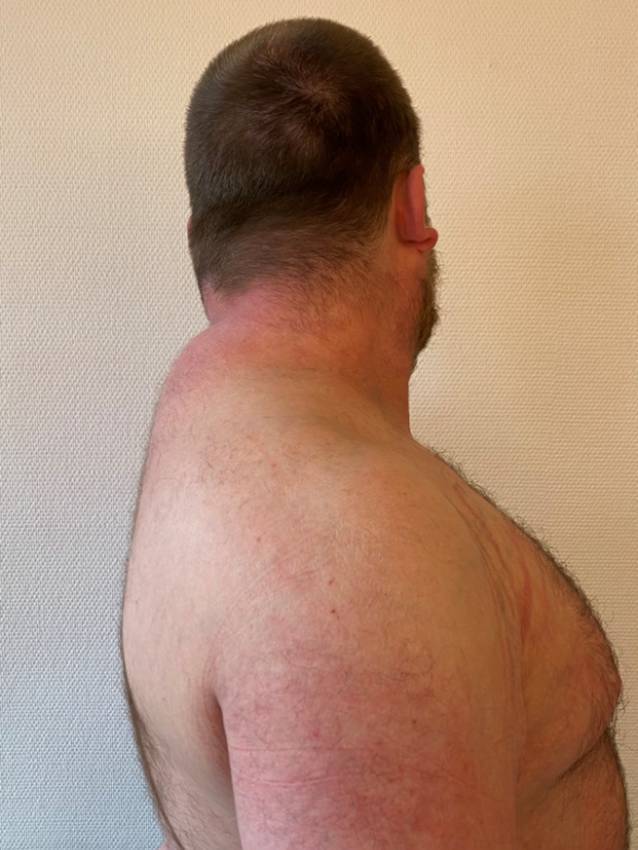

**Figure 2:**
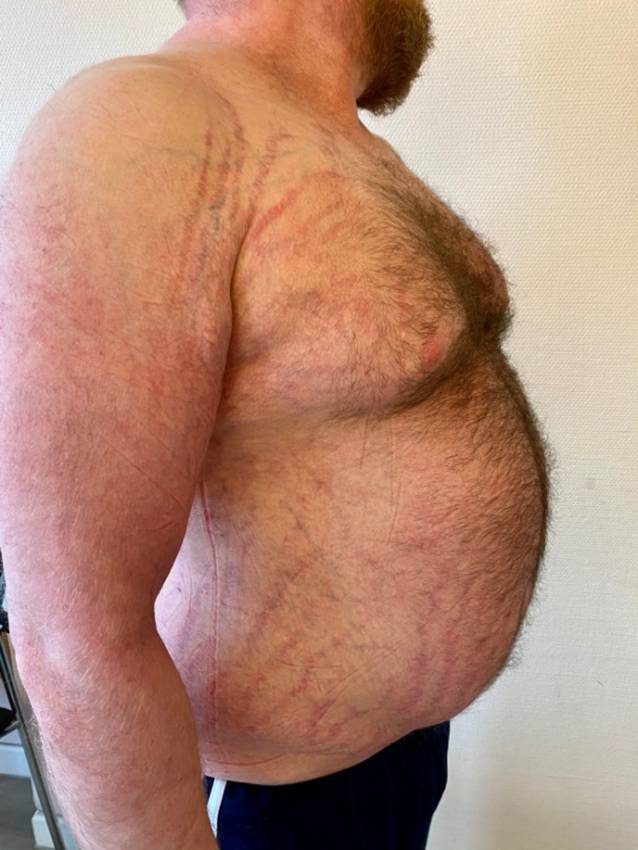

Ein 28-jähriger Patient in adipösem Ernährungszustand (Body-Maß-Index 35,8 kg/m^2^) mit Stammfettsucht und nuchaler Fettgewebsvermehrung („Büffelnacken“) (Abb. 1). Im Nackenbereich, an den Oberarmen, abdominell sowie an den Oberschenkeln zeigen sich unscharf begrenzte Erytheme, teilweise mit Exkoriationen und Lichenifikation im Bereich der Beugeseiten. Die befallene Körperoberfläche („body surface area“ [BSA]) beträgt ca. 10 %. Im Gesicht zeigt sich eine Plethora. Ausgeprägte Striae distensae finden sich am Rumpf und den Oberarmen (Abb. 2). An der rechten Hand lässt sich über den Mittelhandknochen eine Stufenbildung tasten.

## Diagnostik

Im Rahmen der Diagnostik der AD zeigten sich ein stark erhöhtes Gesamt-Immunglobulin E (>5000) sowie ein positiver Phadiatop-Test (Sx1) auf häufige Inhalationsallergene. Bei einem Eczema Area and Severity Index (EASI) von 27 lag eine schwere AD vor, der Dermatologische-Lebensqualitäts-Index (DLQI) wies bei einem Wert von 20 eine schwere Beeinträchtigung der Lebensqualität auf.

Zur Abklärung der Langzeitschäden unter der systemischen Glukokortikosteroidtherapie erfolgte eine weiterführende Diagnostik. Durch wiederholte Blutdruckmessungen konnte eine arterielle Hypertonie ausgeschlossen werden. Laborchemisch zeigte sich der HbA1c mit 6,1 % (Referenz 4,8–5,6 %) erhöht. Zudem konnte ein ausgeprägter Vitamin-D-Mangel mit 7 µg/l nachgewiesen werden (Referenz 30–60 µg/l). Weitere osteologische Laborparameter (Kalzium, Phosphat, alkalische Phosphatase [AP], knochenspezifische AP, γ‑Glutamyltransferase, Kreatinin, Osteocalcin und Desoxypyridinolin Crosslinks im Urin) zeigten sich unauffällig.

Die Knochendichtemessung ergab eine spinal betonte Osteoporose mit einem T‑Score der Wirbelsäule von −2,8 (Referenz T kleiner als −2,5 Standardabweichungen).

Das Röntgenbild der rechten Hand zeigte eine subakute Schaftfraktur des Os metacarpale IV und V. In der Sonographie des Abdomens konnte eine Steatosis hepatis nachgewiesen werden.

Die ophthalmologische Mitbeurteilung ergab einen Normalbefund des linken Auges sowie die bereits bekannte Netzhautablösung des rechten Auges.

## Therapie und Verlauf

Im Rahmen des stationären Aufenthaltes wurde die Prednisolon-Therapie mit 20 mg pro Tag fortgeführt. Vor dem Hintergrund der langjährigen Steroidbehandlung erfolgte unter endokrinologischer Mitbeurteilung die Festlegung eines Ausschleichplans. Dieser sah eine Reduktion von Prednisolon um jeweils 2,5 mg im Abstand von 3 Wochen vor. Zusätzlich solle bei einer Dosis von 5 mg Prednisolon ein ACTH(adrenocorticotropes Hormon)-Kurztest zur Untersuchung der endokrinen Funktion der Nebennierenrinde erfolgen. Aufgrund der Gefahr einer Addison-Krise bei zu schneller Reduktion erfolgte eine ambulante endokrinologische Anbindung.

Bei schwerer AD wurde die leitliniengerechte Systemtherapie mit Dupilumab 600 mg als Initialdosis, gefolgt von 300 mg als Erhaltungsdosis subkutan alle 14 Tage eingeleitet. Eine Lichttherapie mittels UVB-311 nm musste nach 2 Sitzungen mit einer Dosis von jeweils 0,2 J/cm^2^ aufgrund des Auftretens einer Dermatitis solaris beendet werden.

Im Rahmen des osteologischen Behandlungsplans erfolgte eine Substitution von Vitamin D_3_ mittels 20.000 IE 1‑mal täglich über 2 Wochen sowie eine im Anschluss 2‑mal wöchentliche Gabe. Es ist auf eine Aufnahme von 1000 mg Kalzium über die Nahrung zu achten, die im Falle einer nicht ausreichenden Zufuhr durch eine additive Gabe von Calciumglukonat/-citrat ergänzt werden sollte. Durch die Kollegen der Osteologie wurde die Indikation zur spezifischen antiresorptiven Therapie gestellt, die nach erneuter Evaluation des Vitamin D-, Kalzium- und Knochenstoffwechsels unter optimierter Basistherapie vorgesehen ist. Eine engmaschige osteologische Anbindung wurde initiiert. Die subakute Schaftfraktur des Os metacarpale IV und V wurde konservativ mittels Orthese versorgt. Bei beginnendem Steroiddiabetes erfolgte zudem eine Diätberatung mit Aufklärung über eine gesunde Ernährung sowie geeignetes Bewegungsverhalten.

## Diskussion

In den letzten Jahren ließ sich ein rasanter Fortschritt für die Behandlung von Patienten mit schwerer AD verzeichnen. Neben den bereits zugelassenen Biologika Dupilumab (Interleukin[IL]-4-IL-13-Rezeptor-Antikörper) und Tralokinumab (IL-13-Antikörper) sowie den oralen Januskinase(JAK)-Inhibitoren Baricitinib (JAK1/JAK2) und Upadacitinib (JAK1) stehen zahlreiche weitere Systemtherapeutika kurz vor Markteinführung und werden das Therapiespektrum der AD zusätzlich erweitern [1, 6].

Dennoch haben noch nicht alle Patienten Zugang zu diesen modernen Systemtherapien, wie dieser Fallbericht über einen jungen Patienten unter Langzeitbehandlung mit systemischen Glukokortikosteroiden verdeutlicht.

In Tab. 1 sind die häufigsten Nebenwirkungen einer langjährigen Therapie mit Glukokortikosteroiden aufgelistet [3]. Der Patient wies bei einem Alter von 28 Jahren bereits zahlreiche dieser Nebenwirkungen auf.

**Table 1:** 

Nebenwirkung	Häufigkeit
Arterielle Hypertonie	>30 %
Knochenfrakturen/Osteoporose	21–30 %
Übelkeit/Erbrechen/andere gastrointestinale Beschwerden	1–5 %
Herzerkrankungen	4 %
Katarakt	1–3 %
Diabetes mellitus Typ 2/hyperglykämische Stoffwechsellage	Bis zu 4‑fach häufiger
Schlafstörungen	Keine Angabe

Die Leitlinie für die Behandlung der AD empfiehlt den Einsatz systemischer Glukokortikosteroide lediglich in Ausnahmefällen im Rahmen einer akuten Exazerbation bei expliziter Ablehnung einer Langzeittherapie [4, 5]. Das Management im Rahmen der Versorgung von Patienten mit langjähriger Einnahme von Glukokortikosteroiden ist – wie hier aufgezeigt – komplex und verlangt ein interdisziplinäres Vorgehen.

Leider ist bis heute der Einsatz systemischer Glukokortikosteroide bei Patienten mit AD keine Seltenheit. Gemäß den Daten des deutschen Neurodermitis-Registers TREATgermany erhielt bereits mehr als die Hälfte der Patienten mit schwerer AD orale Glukokortikoide [2]. Insbesondere vor dem Hintergrund der zahlreichen Innovationen für die AD muss es das Ziel sein, möglichst allen Patienten mit einer schweren AD eine leitliniengerechte, moderne Systemtherapie zukommen zulassen. Hierbei kommt der Information von Ärzten und Patienten über die neuen Behandlungsoptionen eine große Bedeutung zu.

## Fazit für die Praxis

- Die Zulassung moderner Systemtherapien erlaubt heute auch für die schwere atopische Dermatitis eine sehr wirksame und zudem auch sichere und gut verträgliche Behandlung.
- Eine Anwendung systemischer Glukokortikosteroide bei atopischer Dermatitis ist nur in Ausnahmefällen und immer nur über kurze Dauer angezeigt.
- Im Rahmen der Diagnostik und Versorgung von Patienten mit langjähriger Einnahme von Glukokortikosteroiden ist ein interdisziplinäres Vorgehen von Bedeutung.
